# Systemic Treatment for Metastatic Biliary Tract Cancer: State of the Art and a Glimpse to the Future

**DOI:** 10.3390/curroncol29020050

**Published:** 2022-01-27

**Authors:** Alessandro Rizzo, Angela Dalia Ricci, Antonio Cusmai, Silvana Acquafredda, Giuseppe De Palma, Giovanni Brandi, Gennaro Palmiotti

**Affiliations:** 1 Struttura Semplice Dipartimentale di Oncologia Medica per la Presa in Carico Globale del Paziente Oncologico “Don Tonino Bello”, I.R.C.C.S. Istituto Tumori “Giovanni Paolo II”, Viale Orazio Flacco 65, 70124 Bari, Italy; a.cusmai@oncologico.bari.it (A.C.); s.acquafredda@oncologico.bari.it (S.A.); g.palmiotti@oncologico.bari.it (G.P.); 2 Department of Experimental, Diagnostic and Specialty Medicine, S. Orsola-Malpighi University Hospital, 40138 Bologna, Italy; dalia.ricci@gmail.com (A.D.R.); giovanni.brandi@unibo.it (G.B.); 3 Institutional BioBank, Experimental Oncology and Biobank Management Unit, IRCCS Istituto Tumori “Giovanni Paolo II”, Viale Orazio Flacco 65, 70124 Bari, Italy; g.depalma@oncologico.bari.it

**Keywords:** biliary tract cancer, cholangiocarcinoma, chemotherapy, FGFR2, IDH1, immunotherapy

## Abstract

Recent years have seen some breakthroughs in the therapeutic landscape of advanced biliary tract cancer (BTC). Firstly, a better understanding of the molecular background of BTC has led to important improvements in the management of these hepatobiliary malignancies, with the advent of targeted agents representing an unprecedented paradigm shift, as witnessed by the FDA approval of pemigatinib and infigratinib for FGFR2-rearranged and ivosidenib in IDH1-mutant cholangiocarcinoma. In addition, several novel treatments are under assessment, including immune checkpoint inhibitors and combination chemotherapies. In the current review, we provide an overview of systemic treatment for metastatic BTC, summarizing recent clinical data on chemotherapy as well as the main results of targeted therapies and immunotherapy.

## 1. Introduction

Biliary tract cancers (BTCs) include a heterogeneous group of aggressive and invasive hepatobiliary malignancies, encompassing intrahepatic cholangiocarcinoma (iCCA), perihilar cholangiocarcinoma (pCCA), distal cholangiocarcinoma (dCCA), gallbladder carcinoma (GBC), and ampulla of Vater cancer (AVC) [1,2]. According to the anatomical location within the biliary tree, iCCA occur within the liver parenchyma, while dCCA and pCCA, commonly grouped together under the category of extrahepatic cholangiocarcinoma (eCCA), originate outside the liver [3,4]. Several risk factors have been traditionally associated with the onset of BTC, including liver cirrhosis, hepatolithiasis, hepatitis B and C infection, primary sclerosing cholangitis (PSC), and liver fluke infections (such as *Opisthorchis viverrini* and *Clonorchis sinensis*) [5,6]; of note, these risk factors also reflect the epidemiological differences among the incidence of different BTC forms worldwide, as in the case of hepatobiliary flukes in Southeast Asia [7,8].

Radical surgery with negative margins represents the cornerstone of curative treatment [9,10]; however, patients with early-stage disease are commonly asymptomatic, and unfortunately, only a minority of BTCs are diagnosed with resectable disease, while most of the cases present with locally advanced or metastatic BTC [10]. Despite this, combination chemotherapy with cisplatin–gemcitabine remains the current standard-of-care first-line therapy; recent years have seen the advent of novel treatments in the BTC landscape, including targeted therapies such as pemigatinib, infigratinib, and ivosidenib [11,12,13,14]. In addition, several novel treatments, such as immune checkpoint inhibitors (ICIs), as monotherapy or in combination with other anticancer agents, are under assessment and have the potential to modify the therapeutic scenario of these malignancies [15,16,17,18]. 

In the current review, we provide an overview of systemic treatment for metastatic BTC, summarizing recent clinical data on chemotherapy, targeted therapies, and immunotherapy in this setting. 

## 2. Cytotoxic Chemotherapy

More than 10 years after the publication of the ABC-02 phase III study, cisplatin–gemcitabine chemotherapy still represents the first-line standard-of-care treatment for patients with locally advanced unresectable or metastatic BTC [19,20]. According to the results of this landmark trial published by Valle and colleagues, the combination of cisplatin plus gemcitabine reported longer median overall survival (OS) (11.7 months versus 8.1 months, Hazard Ratio [HR] 0.64, 95% Confidence Interval [CI] 0.52–0.80; *p* < 0.001) and median progression-free survival (PFS) (8.0 months versus 5.0 months) compared with gemcitabine alone [21]. Similarly, the phase II BT22 trial highlighted a similar benefit provided by cisplatin–gemcitabine over gemcitabine monotherapy in Asian patients [22]. Over the last decade, several combination chemotherapies have been investigated to enhance the efficacy of first-line chemotherapy, such as the triplet-agent regimen including cisplatin, gemcitabine, and nab-paclitaxel, with this schedule reporting encouraging clinical outcomes [23,24]. In particular, the median OS and PFS were 19.2 months and 11.8 months, respectively, and the results of the confirmatory phase III trial comparing the reference doublet versus cisplatin–gemcitabine plus nab-paclitaxel are awaited [25]. 

However, most patients experience disease progression following front-line chemotherapy and receive second-line treatment [26,27]. During 2021, the presentation and publication of two potentially practice-changing clinical trials aimed at evaluating second-line chemotherapy in BTC. Among these, the ABC-06 trial compared active symptom control (ASC) plus modified FOLFOX (fluorouracil, folinic acid, and oxaliplatin) (mFOLFOX) versus ASC alone (including biliary drainage, antibiotics, analgesia, etc.) in BTC patients experiencing disease progression following standard cisplatin–gemcitabine [28,29]. According to the results of the study, Lamarca and colleagues observed a statistically significant survival benefit in BTC patients receiving mFOLFOX plus ASC compared to ASC alone, and even though absolute differences in median OS were overall modest (6.2 months and 5.3 months in mFOLFOX plus ASC and ASC alone, respectively), differences in survival rate at 6 and 12 months were clinically meaningful (50.6% versus 35.5% and 25.9% and 11.4%, respectively) [28,29]. Following the results of the ABC-06 phase III trial, the updated National Comprehensive Cancer Network (NCCN) guidelines and the third edition of Japanese guidelines recommend mFOLFOX chemotherapy as the preferred second-line treatment option [30,31]. Conversely, guidelines from the European Society for Medical Oncology (ESMO) have not yet been updated.

Similarly, the recently published South Korean NIFTY phase IIb trial comparing liposomal irinotecan (nal-IRI) plus 5-fluorouracil/leucovorin versus 5-fluorouracil/leucovorin alone showed a survival benefit in BTC patients treated with the doublet [32]. At a median follow-up of 11.8 months, the median PFS for patients receiving liposomal irinotecan plus 5-fluorouracil/leucovorin was 7.1 months compared with 1.4 months for 5-fluorouracil/leucovorin alone, with 44% reduction in the risk of disease progression (HR 0.56; 95% CI, 0.39–0.81). Regarding highly pretreated patients, few data are available regarding third-line chemotherapy; few studies have explored the role of systemic chemotherapy in the third line setting due to several reasons, including the lack of consensus regarding second-line therapy before ABC-06 and NIFTY and the proportion of patients deemed eligible to third- or later-line treatment. Thus, the clinical decision regarding third-line chemotherapy in metastatic BTC remains challenging and is pondered according to several elements, including response to previous treatments, performance status, quality of life, and the patient’s motivation [33].

## 3. Targeted Therapies

**Figure 1:**
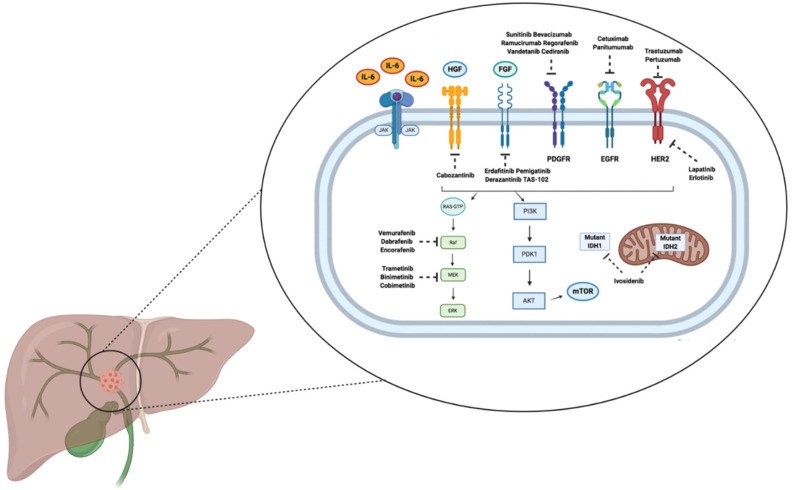
Schematic figure summarizing the main signaling pathways and targeted therapies currently under assessment in biliary tract cancer. Abbreviations: AKT: protein kinase B; EGFR: epidermal growth factor receptor; FGF: fibroblast growth factor; HER2: epidermal growth factor receptor 2; HGF: hepatocyte growth factor; IL-6: interleukin 6; IDH: isocitrate dehydrogenase; JAK: Janus kinase; mTOR: mammalian target of rapamycin; PDGFR: platelet-derived growth factor receptor; PDK1: phosphoinositide-dependent kinase-1; PI3K: phosphoinositide 3-kinase.

The recent advent of genomic sequencing has led to the delineation of the genetic landscape of BTC, suggesting remarkable differences between iCCA, eCCA, GBC, and AVC [34,35,36]. For example, KRAS, ERBB2, and AT-rich interactive domain B (ARID1B) mutations occur more commonly in eCCA and GBC, while fibroblast growth factor receptor 2 (FGFR2) fusions or rearrangements and isocitrate dehydrogenase 1 and 2 (IDH1, IDH2) mutations are nearly exclusive of intrahepatic forms (Figure 1) [37,38,39,40]. Several targeted agents have been developed in this setting: herein, we will discuss the recently approved drugs and the most promising agents in BTC management.

### 3.1. FGFR2 Inhibitors

Several genomic studies have shown that FGFR2 aberrations are detected in approximately 15–20% of iCCAs [41,42]. FGFRs represent tyrosine kinase receptors, with FGFR2 being involved in the upregulation of RAS, JAK, and PI3K/mTOR pathways; thus, FGFR2 aberrations play a role in modifying processes of cellular migration, angiogenesis, proliferation, and survival [43,44]. In the last decade, a wide number of agents targeting FGFR isoforms have been investigated in iCCA patients, such as infigratinib, pemigatinib, derazantinib, erdafitinib and, more recently, futibatinib (Table 1) [45,46]. 

The pan-FGFR tyrosine kinase inhibitor (TKI) infigratinib (or BGJ398) was initially assessed in a phase I basket clinical trial including three CCAs harbouring FGFR2 aberrations, with all patients experiencing stable disease [47]. Subsequently, a phase II trial evaluated the role of infigratinib in 61 gemcitabine–refractory CCA patients with FGFR2 gene fusions, amplifications, or mutations [48]. The overall response rate (ORR) and disease control rate (DCR) were 19% and 83%, respectively, in the subgroup of CCAs with FGFR2 gene fusions; the most commonly reported adverse events included fatigue, hyperphosphatemia, alopecia, and stomatitis. The mature results of this single-arm, phase II study have been recently published by Javle and colleagues, where infigratinib reported a median PFS and OS of 7 and 12 months, respectively [49]. 

The highly selective FGFR1-3 TKI pemigatinib (INCB054828) was firstly evaluated in a basket trial showing partial response in a CCA patient harbouring FGFR2-CCDC6 fusion [50]; no responses were reported in other FGFR aberrations. In the multicentre, single-arm, open-label, multicohort FIGHT-202 trial, previously treated metastatic CCA patients with or without FGFR genetic aberrations received pemigatinib (13.5 mg orally once daily, on days 1–14 of 21-day cycles) [51]. The trial included 107 patients with FGFR2 fusions/rearrangements, 20 with other FGF/FGFR aberrations, and 18 CCA patients without alterations; following a median follow-up of 17.8 months, ORR was observed in 35.5% (38/107) of patients harbouring FGFR2 gene fusions and/or rearrangements, with three cases of complete response and a median duration of treatment of 7.2 months. In the same group, median PFS was 6.9 months and median OS 21.1 months [51,52]. On the contrary, no responses were reported in the other two cohorts of CCA patients; median PFS was 2.1 months and 1.7 months in patients harbouring other FGF/FGFR alterations and in FGFR wild type, with median OS of 6.7 months and 4.0 months in the same cohorts, respectively. Mature efficacy and safety data have been recently presented [53]; according to these results, the median OS of patients with FGFR2 gene fusions and/or rearrangements was 17.5 months (95% CI, 14.4–22.9), reinforcing primary data. In terms of side effects, hyperphosphatemia, arthralgia, and stomatitis were commonly reported, similarly to what was observed in previous trials assessing FGFR inhibitors. Following the results of FIGHT-202, the United States Food and Drug Administration (FDA) granted accelerated approval to pemigatinib for pretreated patients with metastatic CCA harbouring FGFR2 fusions/rearrangements following positive FoundationOne^®^ CDX (Foundation Medicine, Inc., Cambridge, MA, USA) test (Table 2). The results of the ongoing FIGHT-302 trial comparing the efficacy and safety of pemigatinib versus cisplatin–gemcitabine in treatment-naïve patients are highly awaited [54]. 

The pan-FGFR inhibitor derazantinib (ARQ087) was firstly evaluated in a phase I/II study (AR087-101) enrolling 29 CCA patients with FGFR2 gene fusion [55]; most of the patients had experienced disease progression following at least one systemic treatment (*n* = 27), while two cases of treatment-naïve CCA were included. According to the results of this study, ORR and DCR were 20.7% and 82.8%, respectively, with a median PFS of 5.7 months [56]. Another pan-FGFR inhibitor, erdafitinib (JNJ-42756493), was evaluated in a phase I trial, reporting an ORR of 27.3% and a median duration of response of 11.4 months in CCA patients with FGFR mutations or gene fusions [57]. 

Lastly, another promising agent is futibatinib (TAS-120), an irreversible, highly selective pan-FGFR inhibitor able to overcome resistance to ATP-competitive inhibitors [58,59]. In the first dose-escalation phase I trial including metastatic solid tumors with FGFR aberrations, all iCCAs (*n* = 3) achieved partial response [60]; following the results of this trial, the FOENIX-CCA2 single-arm, multicenter, phase II study enrolled 67 pretreated iCCA patients with FGFR2 gene fusions or rearrangements [61]. According to the results of this study, after a median follow-up of 11.4 months, partial response was highlighted in 35.8% of patients receiving futibatinib, with a median PFS of 7.2 months [61]. As previously seen with other FGFR inhibitors, commonly observed treatment-related adverse events of futibatinib included hyperphosphatemia, diarrhea, dry mouth, and alopecia. Given the encouraging signs of activity reported in FOENIX-CCA 2, the ongoing phase III FOENIX-CCA3 clinical trial is comparing futibatinib versus cisplatin–gemcitabine as a first-line treatment for locally advanced unresectable or metastatic iCCA patients harboring FGFR2 gene fusions or rearrangements.

### 3.2. IDH Inhibitors

IDH mutations have been reported in approximately 15% of all cases of iCCA, while these genetic aberrations represent an extremely rare finding in the other BTC subgroups, such as eCCA and GBC [62,63]. From a biological point of view, IDH mutations hesitate in increased IDH1/2 activity, causing changes in cell metabolism and subsequent accumulation of tumour metabolite 2-hydroxyglutaric acid (2-HG) [64,65]. In turn, 2-HG impairs physiological cell differentiation and enhances tumorigenesis via several effects on DNA methylation and chromatin structure [66,67]. Ivosidenib, enasidenib, and other IDH1/IDH2 inhibitors have been recently tested in CCA patients, with some of these agents already reporting important results in other malignancies harbouring IDH mutations. 

Firstly, ivosidenib (AG-120) was tested in a phase I clinical trial enrolling 73 IDH-mutated CCA patients, 5% of which achieved partial response; median PFS and OS were 3.8 months (95% CI, 3.6–7.3) and 13.8 months (95% CI, 11.1–29.3), respectively [68]. Most commonly reported treatment-related adverse events included fatigue, nausea, diarrhoea, and abdominal pain; since the maximum tolerated dose was not reached, the trial selected 500 mg as the recommended dose.

More recently, the phase III ClarIDHy trial randomized 185 previously treated, IDH-mutant CCA patients to ivosidenib or matched placebo, with patients receiving placebo that were allowed to crossover to ivosidenib following radiographic progression [69]. The median PFS was 2.7 months in patients treated with ivosidenib and 1.4 months in those receiving placebo (HR 0.37; 95% CI, 0.25–0.54; *p* < 0.0001). According to the recently presented final results from ClarIDHy, ivosidenib improved median OS by almost three months compared with placebo (10.3 months versus 7.5 months) [70]; although this advantage was not statistically significant, due to crossover, the authors used the RPFST model adjusting OS, and by using this statistical method, the median OS was 5.1 months for patients receiving placebo (HR 0.49; *p* < 0.0001), making this benefit clinically meaningful and statistically significant. Several other IDH inhibitors are currently under assessment in IDH-mutated CCAs, including enasidenib (AG-221) as well as combinatorial strategies including these targeted therapies plus other anticancer agents such as PARP inhibitors.

### 3.3. BRAF Inhibitors

BRAF gene mutations have been highlighted in approximately 5% of BTCs, especially in iCCAs [71,72]. Interestingly, patients harbouring BRAFV600E mutations have a more aggressive clinical course, higher tumour stage at diagnosis, and a greater likelihood of lymph node involvement [73]. As in the case of other BRAF-mutated malignancies, BRAF inhibitor monotherapy has shown short-term responses and rapid onset of treatment resistance in this setting [74]. Thus, combination therapies with BRAF inhibitors plus MEK inhibitors have been tested: the randomized, open-label, single-arm, multicenter ROAR trial recently assessed the combination of dabrafenib and trametinib in BRAFV600E-mutated patients whose disease progressed on gemcitabine-based chemotherapy [75,76]. According to the results of this trial, median PFS and median OS were 9.2 months and 11.7 months in patients receiving dabrafenib plus trametinib [75]; fever, rash, and nausea were the most commonly observed adverse events. 

### 3.4. EGFR Inhibitors

Although activation of EGFR signalling has been reported in CCA patients, EGFR inhibitors such as erlotinib, cetuximab, and panitumumab–as monotherapy or in combination with systemic chemotherapy–reported no OS advantage in several randomized clinical trials, with this lack of benefit also confirmed by some recent meta-analyses [77,78]. Among EGFR inhibitors, HER2-targeted agents have been explored in BTC since approximately 15–20% of GBCs and eCCAs are deemed to present HER2 overexpression or gene amplification; conversely, these alterations are considered quite rare in iCCA [79,80]. Firstly, in a pivotal study including nine GBCs treated with trastuzumab, lapatinib, or pertuzumab, stable disease, partial response, and complete response were observed in three, four, and one case, respectively [81]. On the contrary, no responses were observed in three CCAs [81]. 

More recently, Javle and colleagues reported the results of the BTC cohort of MyPathway HER2 multiple basket trial investigating pertuzumab plus trastuzumab in previously treated patients with HER2 amplification or overexpression [82]. The study highlighted an ORR of 23%, DCR of 51%, and a 1-year OS rate of 50%, with a tolerable safety profile; in addition, the advantage provided by the dual blockade was particularly important in GBC and AVC patients, with a median OS of 14.2 and 17.1 months, respectively.

## 4. Immunotherapy

**Figure 2:**
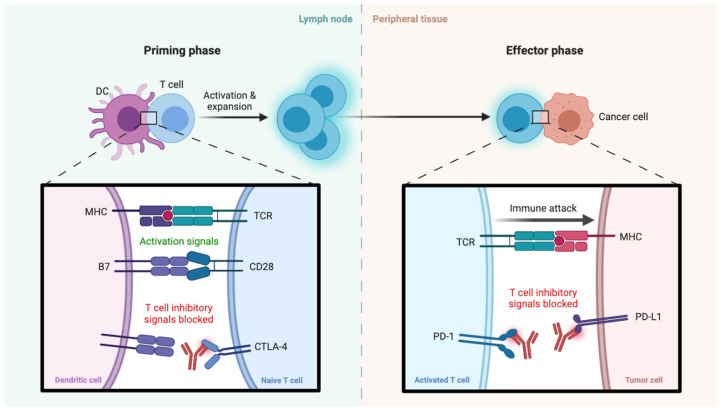
Schematic figure reporting some mechanisms of action of immune checkpoint inhibitors (ICIs) in cancer immunotherapy. On the **left** side of the figure, the immune checkpoint inhibits T-cell activation, while PD-1 inhibitors enhance the immune system response against cancer cells through T cell activation (on the **right**). Abbreviations: CTLA-4: Cytotoxic T Lymphocyte antigen 4; MHC: Major Histocompatibility Complex; PD-1: programmed cell death protein 1; PD-L1: programmed death-ligand 1.

The advent of ICIs has recently made a breakthrough in several hematological and solid tumors, including melanoma, non-small cell lung cancer (NSCLC), hepatocellular carcinoma (HCC), renal cell carcinoma, and urothelial carcinoma [83,84,85,86,87]. These agents are able to boost antitumor activity, leading to enhanced cytotoxicity of T cells and blocking down-regulators of immunity such as programmed cell death protein 1 (PD-1), programmed death-ligand 1 (PD-L1), cytotoxic T-lymphocyte antigen 4 (CTLA-4), and lymphocyte activating-3 (LAG-3) (Figure 2) [88,89,90]. ICIs have also been recently assessed in BTC as monotherapy or in combination with other anticancer agents [91,92].

The PD-1 inhibitor pembrolizumab was assessed in the phase II KEYNOTE-158 and the phase Ib KEYNOTE-028 trials [93]; in these two studies, a small number of pretreated BTC patients whose disease had progressed on standard treatments were enrolled. In the intention-to-treat population, KEYNOTE-158 reported a disappointing ORR of 5.8%, with a median PFS and OS of 2.0 and 7.4 months, respectively; in KEYNOTE-028, the ORR, median PFS, and median OS were 13.0%, 1.8 months, and 5.7 months. However, when the authors stratified their results according to microsatellite instability (MSI) status, MSI-H/dMMR patients highlighted ORR of 40.9%, and median PFS and OS of 4.2 months and 24.3 months, respectively [93]. Another ICI, nivolumab, a human immunoglobulin G4 monoclonal antibody able to block the interaction of PD-1 with PD-L1 and PD-L2, was assessed in metastatic BTC. Firstly, a single-group, multicenter phase II trial on nivolumab monotherapy reported partial response in 10 out of 45 pretreated CCA patients, with 27 subjects achieving stable disease [94]; interestingly, median PFS and OS were 3.68 and 14.24 months, respectively. Secondly, nivolumab has been tested in combination with the reference doublet cisplatin–gemcitabine as a first-line treatment for patients with metastatic disease, reporting a median OS of 15.4 months and a median PFS of 4.2 months [95]; moreover, 11 of 30 patients experienced an objective response. In another phase II trial investigating nivolumab–gemcitabine–cisplatin, the combination therapy reported an ORR of 55.6%, with a median PFS of 6.1 months and median OS of 8.5 months (Table 3) [96]. 

The PD-L1 inhibitor durvalumab, following the practice-changing results reported in other settings and malignancies, has been assessed and is currently under evaluation in BTC [97,98]. In a phase I trial, the combination of durvalumab plus the CTLA-4 inhibitor tremelimumab was explored in advanced solid tumors, including BTCs [99]. Durvalumab monotherapy and durvalumab plus tremelimumab reported median OS of 8.1 months (95% CI, 5.6–10.1) and 10.1 months (95% CI, 6.2–11.4), respectively [99]. Similarly, the dual checkpoint blockade was assessed in a phase II trial assessing nivolumab plus the anti-CTLA-4 agent ipilimumab [100]; according to the results of this study, including 39 BTC patients, the ORR and DCR were 23% and 44%, respectively. The median OS was 5.7 months and the median PFS 2.9 months in patients treated with nivolumab-ipilimumab. 

Although ICIs represent a promising novel frontier in BTC management, several questions remain unanswered. Among these, the lack of validated biomarkers of response represents an important issue since only a proportion of patients benefit from immunotherapy [101,102,103]. For example, the KEYNOTE-028 and KEYNOTE-158 basket trials reported no association between response to ICIs and PD-L1 expression; conversely, the previously cited study published by Kim and colleagues highlighted longer median PFS and higher ORR in PD-L1 positive BTC patients receiving nivolumab. Based on these premises, a deeper understanding of the role of potential biomarkers, including PD-L1 expression, tumor mutational burden (TMB), MSI status, gut microbiota and several others, is fundamental since data regarding their predictive value in BTC patients treated with ICIs are sparse and controversial [104,105,106]. 

## 5. Conclusions

**Figure 3:**
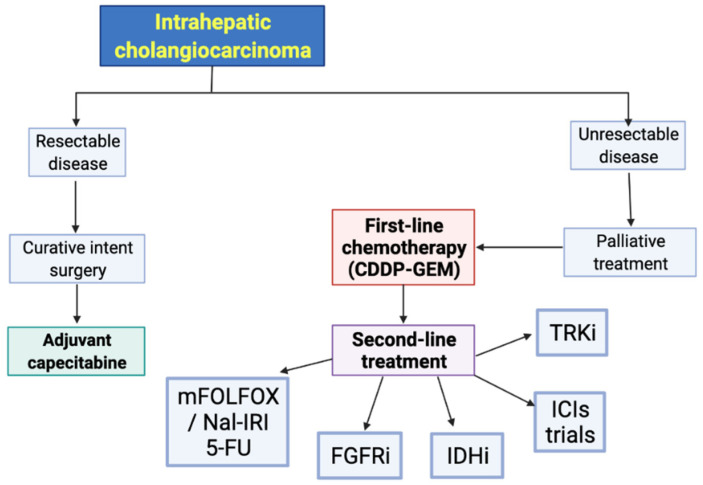
Proposed treatment algorithm for intrahepatic cholangiocarcinoma patients, according to available evidence. Abbreviations: CDDP: cisplatin; FGFR: Fibroblast Growth Factor Receptor inhibitors; GEM: gemcitabine; ICIs: immune checkpoint inhibitors; IDHi: Isocitrate Dehydrogenase inhibitors.

Recent years have seen some breakthroughs in the therapeutic landscape of advanced BTC (Figure 3). Firstly, a better understanding of the molecular background of BTC has led to important improvements in the management of these hepatobiliary malignancies [107,108,109,110]. In fact, the introduction of targeted agents has represented an unprecedented paradigm shift, as witnessed by the FDA approval of pemigatinib and infigratinib for FGFR2-rearranged and ivosidenib in IDH1-mutant CCA. Secondly, the recently published ABC-06 and NIFTY trials have shown an OS benefit in patients receiving mFOLFOX and liposomal irinotecan plus fluorouracil-leucovorin, respectively, as second-line treatment after progression to first-line cisplatin-gemcitabine. In addition, immunotherapy is trying to find its therapeutic niche in BTC patients, where the search for specific histological and molecular predictors of response surely represents one of the current and future challenges. 

## Figures and Tables

**Table 1:** Ongoing clinical trials evaluating FGFR inhibitors in advanced cholangiocarcinoma, according to ClinicalTrials.gov (accessed on 10 January 2022).

Agent	NCT Number	Phase
Infigratinib versus Gemcitabine Cisplatin	NCT03773302	III
Infigratinib	NCT04233567	II
Pemigatinib	NCT04003623	II
Pemigatinib	NCT03822117	II
Pemigatinib versus Gemcitabine Cisplatin	NCT03656536	III
Pemigatinib	NCT04256980	II
Pemigatinib	NCT04258527	I
Gemcitabine Cisplatin plus ivosidenib or pemigatinib	NCT04088188	I
Derazantinib	NCT03230318	II
Derazantinib	NCT04087876	Expanded Access
Erdafitinib	NCT02699606	IIa
Erdafitinib	NCT03210714	II
Erdafitinib	NCT04083976	II
Erdafitinib	NCT02465060	II
Ponatinib	NCT02272998	II
Ponatinib	NCT02265341	II
Futibatinib versus Gemcitabine Cisplatin	NCT04093362	III
Futibatinib	NCT04507503	Expanded Access
Futibatinib	NCT04189445	II
Debio 1347	NCT03834220	II

**Table 2:** Approved agents in FGFR2-positive cholangiocarcinoma.

Agent	Company	Approval
Pemigatinib	Incyte	FDA
Infigratinib	Novartis/QED	FDA EMA

**Table 3:** Summary of main clinical trials evaluating immune checkpoint inhibitors in biliary tract cancer patients with advanced disease.

Treatment Arm	Agents Description	NCT Name	Phase	Setting	Results
Pembrolizumab	Pembrolizumab: PD-1 inhibitor	NCT02628067 (KEYNOTE-158) [93]	II	Second- or later-line	ORR: 5.8% (2.1–12.1) mOS: 7.4 mo (5.5–9.6) mPFS: 2.0 mo (1.9–2.1)
Pembrolizumab	Pembrolizumab: PD-1 inhibitor	NCT02054806 (KEYNOTE-028) [93]	Ib	Second- or later-line	ORR: 13.0% (2.8–33.6) mOS: 5.7 mo (3.1–9.8) mPFS: 1.8 mo (1.4–3.7)
Nivolumab	Nivolumab: PD-1 inhibitor	JapicC- TI-153098 [96]	I	Second- or later-line	ORR: 3.3% (0.7–13.6) mOS: 5.2 mo (4.5–8.7) mPFS: 1.4 mo (1.4–1.4)
Nivolumab	Nivolumab: PD-1 inhibitor	NCT02829918 [94]	II	Second- or later-line	PR: 22% DCR: 59% mOS: 14.2 mo (5.98-not reached) mPFS: 3.7 mo (2.3–5.69)
Durvalumab	Durvalumab: PD-L1 inhibitor	NCT01938612 [99]	I	First- or later-line	DCR: 16.7% mOS: 8.1 mo (5.6–10.1)
Nivolumab plus ipilimumab	Nivolumab: PD-1 inhibitor Ipilimumab: CTLA-4 inhibitor	NCT02923934 (CA209–538) [100]	II	First- or later-line	ORR: 23% DCR: 44% mOS: 5.7 mo (2.7–11.9) mPFS: 2.9 mo (2.2–4.6)
Durvalumab plus tremelimumab	Durvalumab: PD-L1 inhibitor Tremelimumab: CTLA-4 inhibitor	NCT01938612 [99]	I	First- or later-line	DCR: 32.2% mOS: 10.1 mo (6.2–11.4)
Nivolumab plus CisGem	Nivolumab: PD-1 inhibitor	NCT03311789 [95]	II	First-line	ORR: 55.6% DCR: 92.6% mOS: 8.5 mo (5.0–12.5) mPFS: 6.1 mo (3.4–8.2)
Nivolumab plus CisGem	Nivolumab: PD-1 inhibitor	JapicCTI- 153098 [96]	I	First-line	ORR: 36.7% mOS: 15.4 mo (11.8-NE) mPFS: 4.2 mo (2.8–5.6)

CisGem, cisplatin plus gemcitabine combination; CTLA-4, Cytotoxic T-Lymphocyte Antigen 4; DCR: disease control rate; NE: not estimable; ORR, overall response rate; mo: months; mOS, median overall survival; PD-1, programmed death 1, mPFS: median progression-free survival; PR: partial response.
